# Alginate Beads Containing Cerium-Doped Mesoporous Glass and Curcumin: Delivery and Stabilization of Therapeutics

**DOI:** 10.3390/ijms24010880

**Published:** 2023-01-03

**Authors:** Debora Carrozza, Gianluca Malavasi, Erika Ferrari, Maria Cristina Menziani

**Affiliations:** Department of Chemical and Geological Sciences, University of Modena and Reggio Emilia, Via G. Campi 103, 41125 Modena, Italy

**Keywords:** antioxidant properties, composite biomaterials, alginate beads, hydrogels

## Abstract

Cancer is a leading cause of death worldwide, its genesis and progression are caused by homeostatic errors, and reactive oxygen species play a major role in promoting aberrant cancer homeostasis. In this scenario, curcumin could be an interesting candidate due to its versatile antioxidant, anti-inflammatory, anti-tumor, anti-HIV, and anti-infection properties. Nonetheless, the major problem related to its use is its poor oral bioavailability, which can be overcome by encapsulating it into small particles, such as hydrogel beads containing mesoporous silica. In this work, various systems have been synthesized: starting from mesoporous silica glasses (MGs), cerium-containing MGs have been produced; then, these systems have been loaded with 4 to 6% of curcumin. Finally, various MGs at different compositions have been included in alginate beads. In vitro studies showed that these hybrid materials enable the stabilization and effective delivery of curcumin and that a synergic effect can be achieved if Ce^3+^/Ce^4+^ and curcumin are both part of the beads. From swelling tests, it is possible to confirm a controlled curcumin release compartmentalized into the gastrointestinal tract. For all beads obtained, a curcumin release sufficient to achieve the antioxidant threshold has been reached, and a synergic effect of cerium and curcumin is observed. Moreover, from catalase mimetic activity tests, we confirm the well-known catalytic activity of the couple Ce^3+^/Ce^4+^. In addition, an extremely good radical scavenging effect of curcumin has been demonstrated. In conclusion, these systems, able to promote an enzymatic-like activity, can be used as drug delivery systems for curcumin-targeted dosing.

## 1. Introduction

Cancer genesis and progression is caused by homeostatic errors occurring within the tumor microenvironment [1], related or not to genetic mutations, addressing all components of the cancer tissue [2], and implying many alterations, including increased oxidative status. Reactive oxygen species (ROS) play a major role in promoting the aberrant cancer homeostasis, favoring vicious communications between cancer cells and stroma, endothelium, and matrix, thus favoring tumor neo-angiogenesis, matrix degradation, and improper immune infiltrations [3]. Hence, antioxidant therapy is considered as a means to prevent and revert the alteration of the tumor microenvironment.

Curcumin is widely used as a pharmaceutical or nutraceutical in functional foods, food supplements, and medicine [4,5] due to its versatile antioxidant, anti-inflammatory, anti-tumor, anti-HIV, and anti-infection properties [2,3,4]. However, the formulation of curcumin as a therapeutic agent is a difficult task due to its low aqueous solubility and chemical and metabolic instability, resulting in poor oral bioavailability [5,6]. 

Curcumin is rapidly metabolized within the gastrointestinal tract (GIT), which limits its potential beneficial biological effects [7,8,9]. To overcome these limitations, several techniques have been proposed, including encapsulation in a delivery system [10] that consists of small particles (typically comprising lipids, phospholipids, surfactants, and/or biopolymers) [11]; these delivery systems can be designed to improve curcumin’s chemical/biochemical stability and to control its fate within the gastrointestinal tract. 

Previous studies [12] have demonstrated the potential of improving the bioavailability of curcumin by using emulsion-based carriers. In particular, the lipid phase in emulsions can be designed to rapidly digest within the small intestine and form mixed micelles capable of solubilizing and transporting lipophilic bioactive components [12,13]. One of the main limitations of conventional oil-in-water emulsions as drug carriers is that they have only a limited scope in controlling the stability and release of encapsulated bioactive agents since the lipid droplets are coated by a thin layer of emulsifier molecules. Furthermore, lipids droplets can present some limitations concerning the stability of drug encapsulation, whereby important drug leakage can be observed because, at times, a phase demixing and drug expulsion can be induced [14]. These limitations can be overcome by trapping the lipid droplets inside hydrogel beads [15]. 

Hydrogel beads are usually fabricated from food-grade biopolymers, such as proteins and/or polysaccharides [16,17,18]. Among the wide number of different approaches [19] that can be used for encapsulation, protection, and delivery of food-grade bioactive components, the injection–gelation method is one of the simplest and most widely utilized. In this method, a biopolymer solution (typically alginate or κ-carrageenan) containing the bioactive component is injected into another “hardening” solution under conditions that promote the gelation of the injected biopolymer. This procedure results in the formation of a hydrogel bead with the bioactive components trapped inside [20]. The nature of the hydrogel matrix can be designed to improve its physical and chemical stability, control its gastrointestinal track fate, and synergize the beneficial properties by adding other antioxidant agents. It has recently been reported [21] that ionic cross-linking of alginate and mesoporous bio-glasses (MGs) containing various amounts of cerium oxides (Ce^3+^/Ce^4+^) produces excellent candidates for oxidative stress control. 

The encapsulation of MGs inside beads [22] allows not only the development of antioxidant properties but also the improvement of physical and mechanical properties of the beads [23], which became more resistant to dissolution, and, thus, rehydration can be easily performed [24]. The introduction of small amounts of CeO_2_ into silicate glasses (for example in mesoporous glasses, MG) confers antioxidant properties, such as catalase mimetic activity [25]. Catalase is an enzyme that catalyzes the decomposition of hydrogen peroxide (H_2_O_2_) into water and oxygen. Its role is very important because it protects cells from oxidative stress by ROS, of which hydrogen peroxide is part. We recently [26] showed that the ability of these glasses to present catalase mimetic activity is strictly related to the simultaneous presence of Ce^3+^ and Ce^4+^ on the glass [27] surface, and we developed biocompatible Ce-MG/alginate beads able to promote a fast degradation of H_2_O_2_ to reduce the oxidative stress.

The aim of this work is to develop a composite material on the basis of Ce-MG/alginate beads (B-MG) acting as carriers for curcumin. Two mesoporous glasses were synthesized, one with a standard composition (MG) and a second one doped with cerium (MG_5.0Ce), and both were investigated as powders (P). The MG powdery forms were uploaded with curcumin (P-MG_curc5 and P-MG_5.0Ce_curc5) and used to prepare the hydrogel beads (B-MG_curc5 and B-MG_5.0Ce_curc5). We will demonstrate that this delivery system is able to promote an enzymatic-like activity (catalase), thus reducing the concentration of H_2_O_2_, a potent ROS, and potentiate the beneficial effects of curcumin by protecting it through the gastrointestinal tract with a subsequent drug release in the intestinal environment.

## 2. Results

### 2.1. P-MGs and B-MGs Characterization

The compositions of P-MGs and B-MGs were determined using two different methods: alginate and glass content were determined by TG-thermogravimetric analysis and elemental analysis, and curcumin content was determined through UV-Vis quantification.

Table 1 reports the amount of each component for all the samples studied: P-MGs and all the diff B-MGs obtained containing only the bioactive glass (B-MG and B-MG_5.0Ce), both bioactive glass and curcumin (B-MG_curc5, B-MG_5.0Ce_curc5), only curcumin (B-curc), and only the sodium alginate (B).

**Figure 1:**
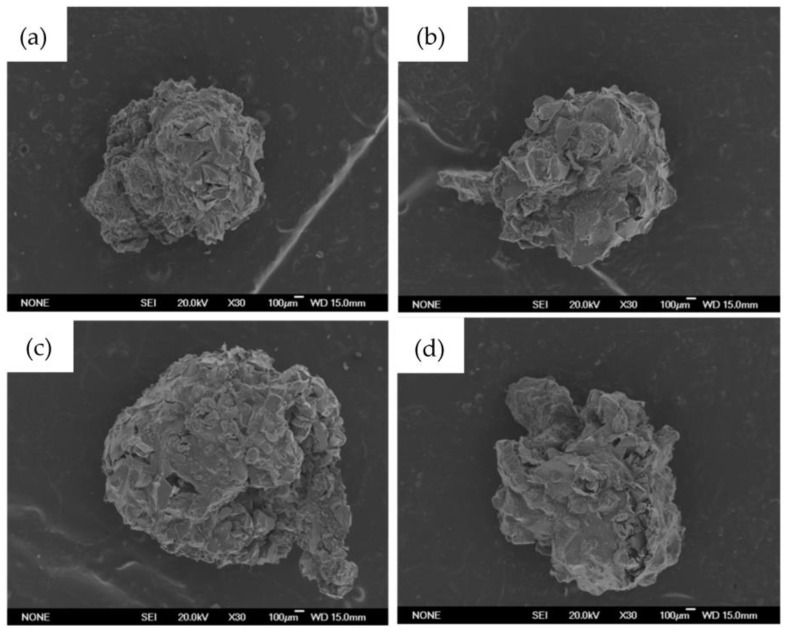
Bead images obtained with scanning electron microscope (SEM). In order: (**a**) B-MG; (**b**) B-MG_5.0Ce; (**c**) B-MG_curc5; and (**d**) B-MG_5.0Ce_curc5.

Figure 1 shows the SEM images of beads containing MBGs powders. The micrographs show that the surface is characterized by a partially homogeneous distribution of the glass powders incorporated and held together in a polymeric matrix of alginate. The presence of volume defects, such as fractures, have been likely formed during the drying process at 60 °C. 

**Figure 2:**
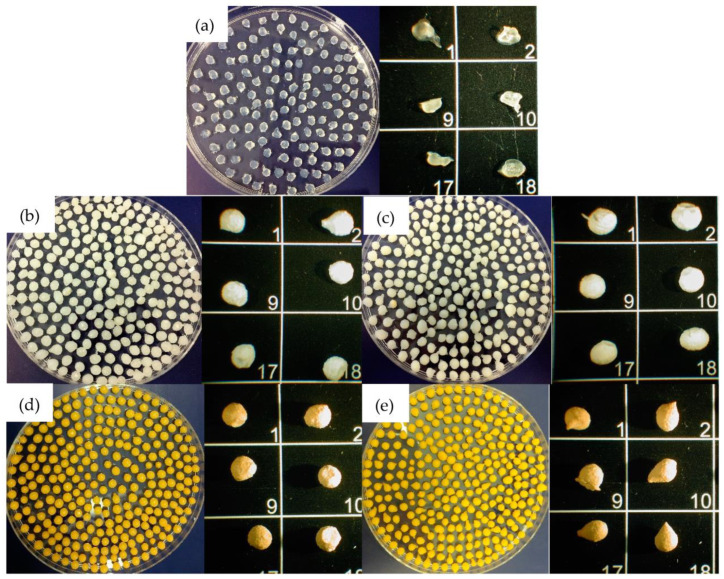
Beads images obtained with optic microscope. In order: (**a**) B; (**b**) B-MG; (**c**) B-MG_5.0Ce; (**d**) B-MG_curc5; and (**e**) B-MG_5.0Ce_curc5. The numbers identify different beads of the same sample.

To evaluate B-MGs dimensions, images with optical microscope have been acquired (Figure 2), and through an image analysis program (ImageJ^®^-win64), dimensions of the beads have been determined using the average of 24 measures for each sample (Table 2).

As it is possible to notice from the data reported in Table 2, beads containing curcumin are slightly larger than those containing only MGs; this evidence could be explained by considering the surface layer made by deposition of curcumin formed during the loading process into MGs.

**Figure 3:**
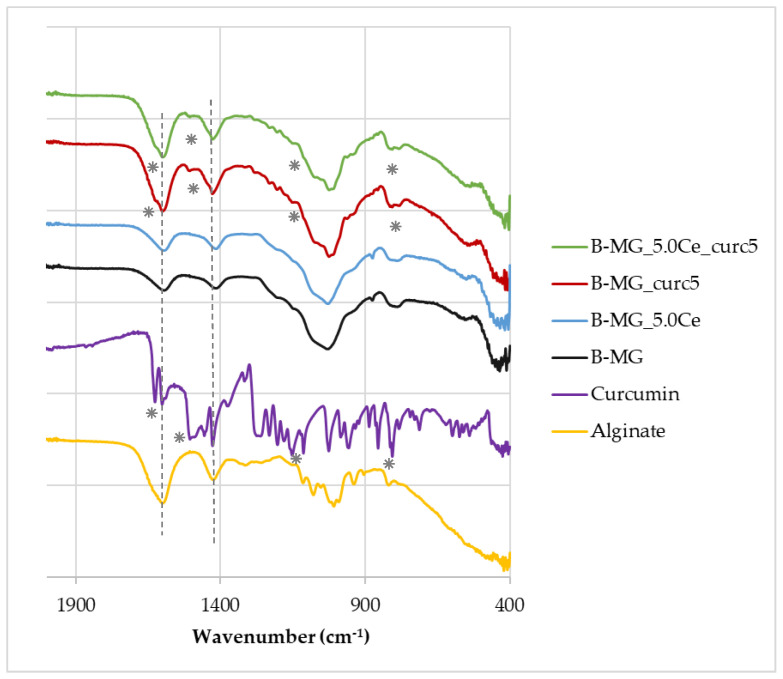
FTIR−ATR spectra of beads, pure alginate, and curcumin. Characteristic peaks of alginate and curcumin are highlighted by dashed lines and asterisks, respectively.

FTIR−ATR spectra (Figure 3) were acquired for all beads and for pure alginate and curcumin to highlight the presence of signals of all components inside the beads. 

The FTIR−ATR spectra show that the characteristic peaks of alginate (dashed lines) at 1419 cm^−1^ and 1601 cm^−1^ [28] are present in all samples, whereas the characteristic peaks of curcumin (asterisks) at 1630 cm^−1^, 1516 cm^−1^, 1093 cm^−1^, and 798 cm^−1^ [29] are present only in B-MG_curc5 and B-MG_5.0Ce_curc5, providing further evidence of the success of curcumin uploaded. 

### 2.2. Swelling Test

**Figure 4:**
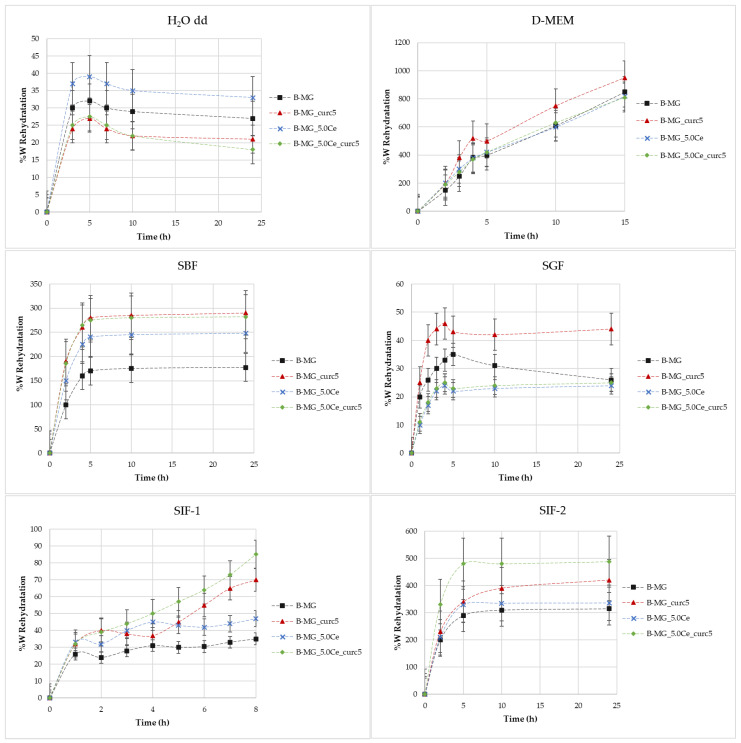
Rate of rehydration (%W) as a function of time in different media.

The rehydration rate [30] (Figure 4) was obtained using gravimetric analysis from the difference between initial mass and the mass calculated after specific time intervals.

Figure 4 shows the rate of rehydration (%W) as a function of time in different media: H_2_O, simulated body fluid (SBF), Dulbecco’s Modified Eagle Medium (D-MEM), and simulated gastric fluid (SGF) and simulated intestinal fluid (SIF) with various buffers.

*Swelling in H_2_O MilliQ*—In these cases, there is no evidence of a high rehydration rate because no values greater than 40% were reached. During the first 6 h, swelling takes place, then a small decline in rehydration rates occurs. This indicates a partial solubilization. 

*Swelling in D-MEM*—In these cases, rehydration rates are extremely high. At 24 h, it was not possible to evaluate the beads’ mass because of their disruption. The maximum time reached was 15 h. In this medium, the rehydration is gradual; the systems rehydrate gradually and become very fragile. In the presence of MGs, inside the structure, the beads show more compactness.

*Swelling in SBF*—Initially, a large swelling was registered, then a plateau was reached. The highest swelling is observed for samples with lower MGs concentrations. This phenomenon is caused by the formation of a hydroxyapatite (HA) layer on the surface of the beads as a direct function of the MGs concentration formed at physiological conditions. 

*Swelling in SGF*—It was demonstrated that the rehydration rate is proportional to the pH value [4]. In this medium, rehydration rates greater than 40% were not observed. 

*Swelling in SIF*—For these tests, two different types of intestinal fluids were employed: SIF-1 (phosphate buffer 0.01 M at pH of 6.8) and SIF-2 (TRIS buffer 1 M at pH 6.8). In SIF-1, no high values were reached, whereas SIF-2 caused elevated swelling. It is probable that this phenomenon is caused by the interaction between TRIS and alginic residuals of alginate chains.

### 2.3. Curcumin Release

Both MGs and beads were studied. For all the samples, the same mathematical model, Higuchi’s model [31], can be applied (Equation (1)). The release has been detected for 24 h, but models must be applied only in the time interval in which the release has significant values; under this condition, the equation is linear.

Q = k · t^½^ (1)

in which Q is the quantity of drug release, t is the time, and k is the diffusion constant. 

The generalization of this model leads to Peppas’ equation [32] (Equation (2)).

Q = k · t^n^ (2)

If n, obtained from the linearization of the plot of % curcumin release vs time, is 0.5, the release is under diffusion control; if it is 1, the release is controlled by the erosion of the system and the swelling of the polymer.

### 2.4. MGs’ Curcumin Release

**Figure 5:**
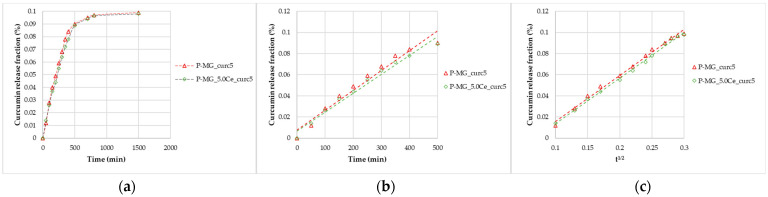
Curcumin release kinetics from P-MGs; (**a**) graph of the curcumin fraction released in the first 24 h compared with the amount contained in 50 mg; (**b**) linearization according to Peppas’ model (n = 1); and (**c**) linearization according to Higuchi’s model.

The studies were performed in SBF (pH 7.2–7.4). At different timing, curcumin concentration was determined by spectrophotometric analysis (Figure 5a), and the SBF solution was replaced with a new one every time. In addition, after 24 h, a slight release was registered. Both models are applied to perform data linearization (Figure 5b,c); Higuchi’s model confirms the diffusion control, while Peppas’ model (n = 1) confirms the erosion/dissolution of the glass matrix.

After 24 h, from 50 mg of P-MGs, a release of approximately 1 µg of curcumin in 5 mL of biological medium was achieved; this amount is comparable to that detected in serum (curcumin level of 0.36 µg/mL) after an intravenous injection of 10 mg/kg given in rats [6].

### 2.5. Beads’ Curcumin Release

The studies were performed in three different biological fluids: SBF, SGF, and SIF (SIF-1 and SIF-2). To confirm the diffusive nature of curcumin release, the same mathematical models described above were applied. 

### 2.6. Release in Simulatd Body Fluid (SBF)

**Figure 6:**
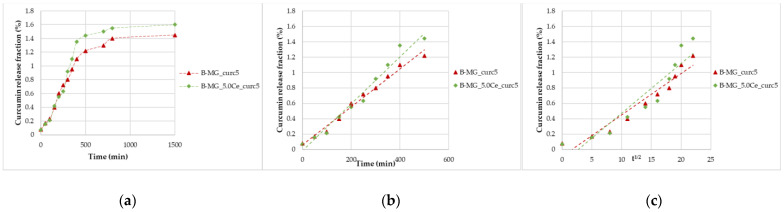
Curcumin release kinetics from B-MGs in SBF; (**a**) curcumin fraction released in the first 24 h for the two samples B-MG_curc5 and B-MG_5.0Ce_curc5; (**b**) linearization according to Peppas’ model (n = 1); and (**c**) linearization according to Higuchi’s model (n = 0.5).

These studies were performed for 24 h (Figure 6a). The same trend seen for MGs was registered, and by applying the two mathematical models, good regressions were obtained (Figure 6b,c). Concerning beads, diffusion and swelling/desegregation occur simultaneously. 

From the collected data, a release of 3.0–3.1 µmol of curcumin per 100 mg of beads was recorded, a higher quantity than the minimum necessary to have a pharmaceutical effect [5,6].

### 2.7. Release in Simulated Gastric Fluid (SGF)

**Figure 7:**
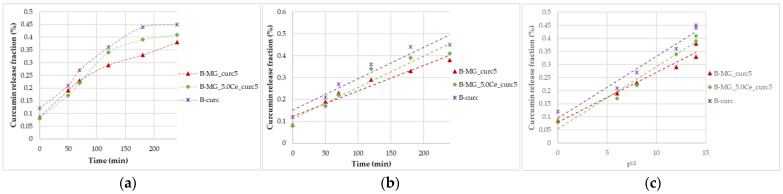
Curcumin release kinetics from B-MGs in SGF; (**a**) curcumin fraction released in the first 24 h for the samples B-MG_curc5 and B-MG_5.0Ce_curc5, and B-curc; (**b**) linearization according to Peppas’ model (n = 1); and (**c**) linearization according to Higuchi’s model.

Simulated gastric fluid (pH = 1.2) allows for the study of the beads’ behavior in an acid environment. Beads were tested for 4 h. All beads showed acid resistance, no significant swelling occurred, and, consequently, no high percentage of curcumin was released (Figure 7).

### 2.8. Release in Simulated Intestinal Fluid (SIF)

**Figure 8:**
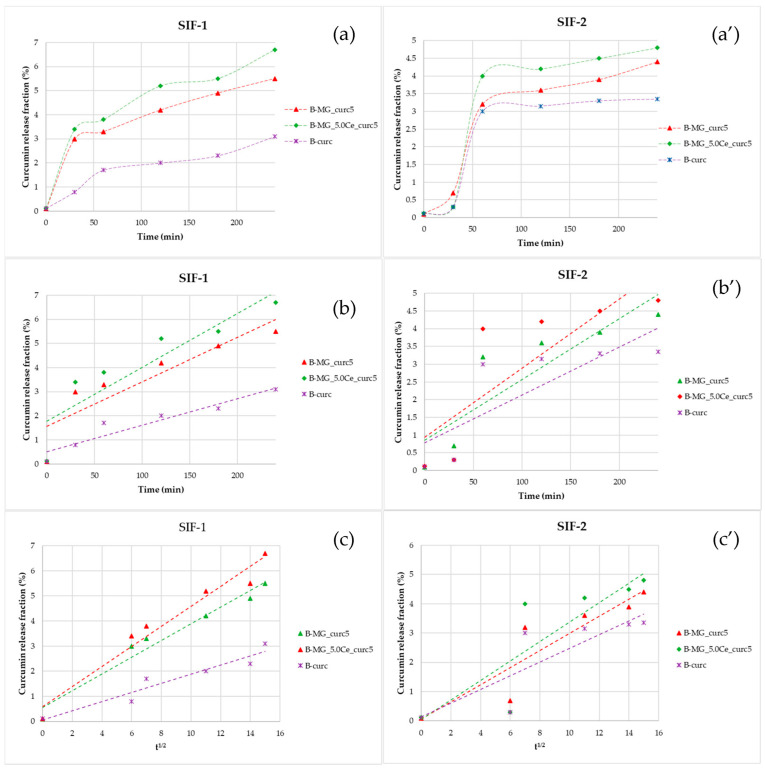
Representations of the kinetics release of the beads in SIF. (**a**,**a′**) Percentage fraction of time-released curcumin; (**b**,**b′**) linearization according to Peppas’ model; and (**c**,**c′**) linearization according to Higuchi’s model.

Studies in SIF were conducted after the incubation in SGF. Comparing the percentage release of curcumin in both simulated fluids, a more gradual release for beads in SIF-1 than in SIF-2 (Figure 8a,a′) was noticeable; thus, a different swelling behavior in different media is observed (Figure 8b,b′,c,c′). During the first 30 min, the release is higher due to the adaptation of the beads to the new environment. 

Linearizing the data collected in SIF-1 highlighted the prevalence of diffusive phenomena. For beads containing MGs, a diffusive release is still prevalent after 4 h. Release in SIF-2 is more gradual: during the first 30 min, no release is registered, but release takes place between 30 and 60 min. The release in this interval is not controlled; in fact, no good results have been obtained, either with Higuchi’s linearization or with Peppas’ model.

### 2.9. SEM Characterization after Soaking in SGF and SIF

**Figure 9:**
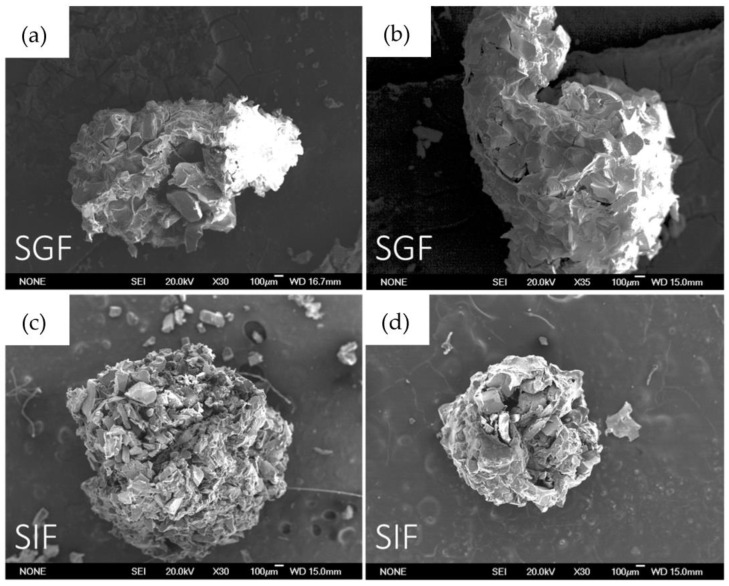
SEM images in order: (**a**) B-MG_curc5 after 4 h soaking in SGF; (**b**) B-MG_5.0_curc5 after 4 h soaking in SGF; (**c**) B-MG_curc5 after 4 h soaking in SIF; and (**d**) B-MG_5.0Ce_curc5 after 4 h soaking in SIF.

To control the external aspect of beads after soaking in SGF and SIF, SEM characterization was performed. As is possible to notice in Figure 9, and as was confirmed by curcumin release, in SGF, the structure of the beads is maintained and only a few cracks are evident. Furthermore, after soaking in SIF, the surface appears damaged, and as was confirmed by curcumin release, the alginate dissolution allows curcumin release in intestinal fluids.

### 2.10. Catalase Mimetic Activity (CMA) Tests

#### CMA Tests of Powders

The enzymatic-like activity of MG_5.0Ce glasses has been demonstrated previously [1]; therefore, we focused on the curcumin effect on the degradation process of peroxide.

**Figure 10:**
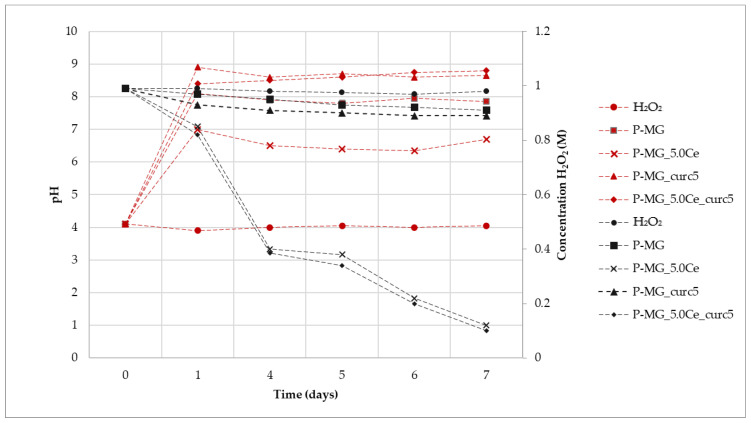
Variation of H_2_O_2_ concentration mediated by samples (black) and variation of pH (red) during the assay time.

As shown in Figure 10, where the variation of H_2_O_2_ concentration vs. time is plotted for the samples at different pH values, the degradation registered for the curcumin-loaded samples is the spontaneous degradation of a H_2_O_2_ solution. The slight increase of CMA for the P-MG_5.0Ce_curc5 sample could be explained by considering the presence of phosphate groups that could influence the activity. Phosphate groups present in MGs stabilize Ce^3+^ ions, forming an insoluble phase of CePO_4_ that blocks the interconversion between Ce^3+^ and Ce^4+^.

Samples containing cerium degrade approximately 90% of H_2_O_2_, whereas samples without cerium, in 7 days, degrade only 10–15% of H_2_O_2_.

The kinetic degradation of each sample was studied by applying mathematical models for first- and second-order reactions (Table 3).

Samples containing cerium show a first-order degradation constant. The presence of Ce^3+^ causes faster degradation of H_2_O_2_; moreover, the degradation depends only on its presence, as shown by the poor R^2^ for H_2_O_2_ due to a very limited degradation. 

For all samples, an increase of pH is registered during the first 24 h due to the release of Ca^2+^ in solution (Figure 9). This effect does not affect the Ce^3+^ catalytic activity, which occurs both at physiological and slightly basic pH (pH of approximately 7 for P-MG_5.0Ce and 8 for P-MG_5.0Ce_curc5).

To demonstrate that the catalytic system Ce^3+^/Ce^4+^ persists over time, samples containing Ce^3+^, which had already been in an incubator for 7 days, were kept in contact with fresh H_2_O_2_ 1 M solution. At the end of the additional 7 days, the titration of H_2_O_2_ was performed, and a degradation rate of 90–95% was registered. 

These systems act as antioxidants (CMA) with a time-dependent activity due to the solubilization of the systems in physiological ambient environments.

### 2.11. CMA Tests of Beads

**Figure 11:**
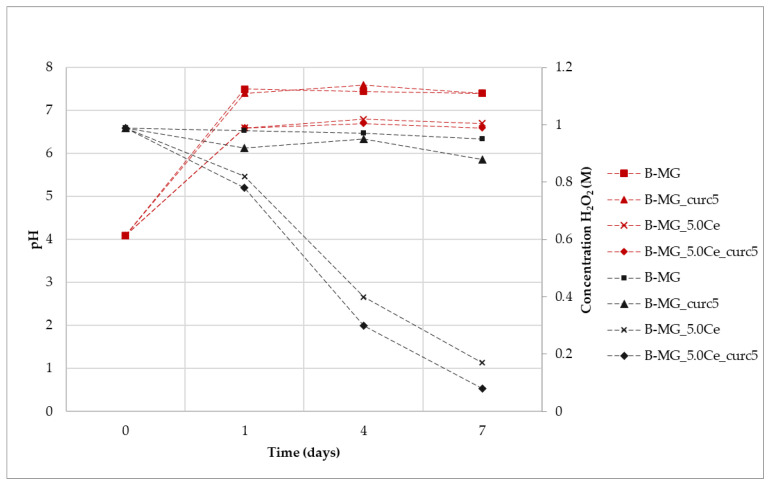
Variation of H_2_O_2_ concentration mediated by samples (black) and variation of pH (red) during the assay time.

The synergic effect on the beads caused by the presence of alginate and curcumin was examined (Figure 11). In general, a trend similar to that which was previously reported in Figure 8 is observed; therefore, the degradation process of peroxide is not affected by the presence of both alginate and curcumin.

Kinetic degradation was studied by applying mathematical models for first- and second-order reactions. Accordingly, to results collected for P-MGs, beads samples containing cerium with or without curcumin follow a first-order kinetic, while beads containing only MG with or without curcumin follow a second-order kinetic (Table 4).

For all the suspensions, the pH values during the time were recorded (Figure 10). An increase of pH was registered for all samples due to the presence of Ca^2+^ in the solution, according to previous results. It is worth noticing that the pH value of samples without cerium is higher in comparison with the samples containing it. This can be explained by the interactions among MG, alginate, and curcumin, which are enforced by the presence of cerium. This phenomenon causes a reduction in the exchange between Ca^2+^ and protons, thus exhibiting a lower pH value. After 24 h, the pH value stabilizes at basic pH, but Ce^3+^ activity persists. Therefore, as was the case for the beads, the catalytic activity also occurs at physiological pH.

### 2.12. Antioxidant Activity, DPPH Test

**Figure 12:**
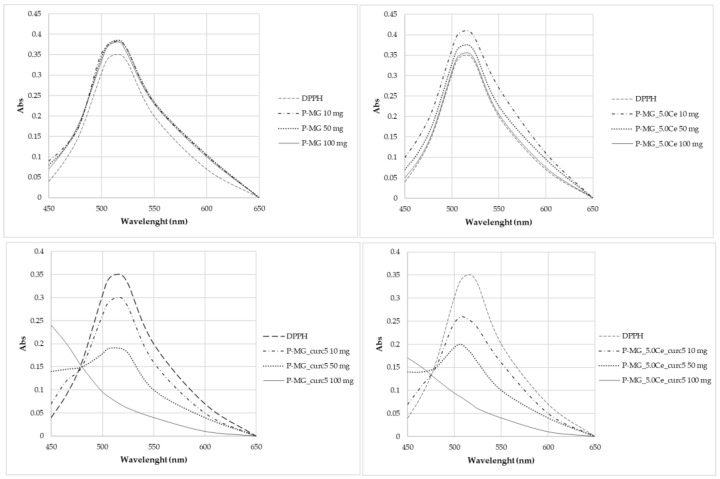
UV-Vis absorption spectra after 2 h contact with DPPH.

The antioxidant activity of curcumin has been evaluated by analyzing the decrease in the UV-Vis adsorption of DPPH in correspondence with the maximum. The spectra in Figure 12 show that degradation occurs only for samples containing curcumin, in which the adsorption of radicals is extinguished. In particular, the higher the curcumin concentration, the higher the depletion.

**Figure 13:**
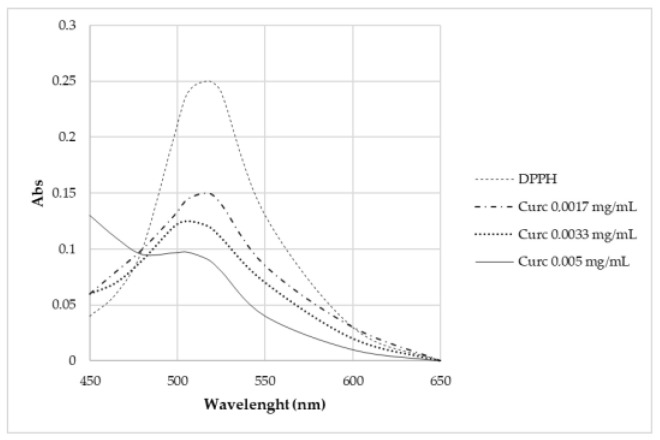
UV-Vis absorption spectra of samples kept in contact for 2 h with DPPH.

Curcumin expresses activity as a radical scavenger [2]. For this region, this work also investigated the concentration at which curcumin does not cause the complete degradation of DPPH (Figure 13). Different concentrations were studied: 5·10–3 mg/L, 3.3·10–3 mg/L, and 1.7·10–3 mg/L. None of these solutions brought about the complete degradation of DPPH in 2 h time.

Samples antioxidant activity was calculated using a formula proposed by Gulcin (Equation (3)) [3,29]:

(3) $$Radical\;Scavenging\;Effect\;\left(RSE\right)\;\%=\left(1-\frac{A\;sample}{A\;reference}\right)\cdot 100$$

From the *radical scavenging effect* (*RSE*%) calculation (Table 5), the significant action of curcumin on the depletion of radicals can be highlighted, whereas no activity is observed for P-MGs. Moreover, the action against radicals is attenuated in P-MG_5.0Ce_curc5 with respect to P-MG_curc due to the controlled release of curcumin when cerium is present.

### 2.13. DPPH Degradation Kinetic

Table 6 lists the linear equations and related parameters for the kinetic study of antioxidant power in the DPPH test.

All samples show a second-order kinetic; for samples with no curcumin, this depends strictly on depleting agents present on the surface of the solids.

**Figure 14:**
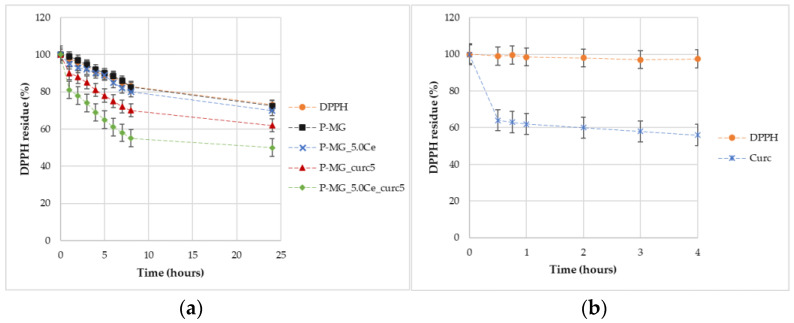
Graphic representation of the percentage of DPPH remaining over time for samples (**a**) and for curcumin effect (**b**).

Through percentage degradation analysis of DPPH radicals (Figure 14a), it was possible to observe that samples containing curcumin depleted a higher quantity of radicals in comparison with the parallel samples without curcumin. Moreover, the synergic effect of the couple cerium–curcumin is also demonstrated. The degradation activity of curcumin is higher during the first hour (Figure 14b); after this time, the action slows down.

## 3. Discussion

The synergistic application of various experimental techniques applied in this study demonstrates that the composite material based on Ce-MG/alginate beads (B-MG) is a suitable drug delivery system for curcumin.

The curcumin molecule, after the loading, shows the characteristic adsorbing peak in the UV-Vis spectrum performed on solid-state, indicating that its integrity is maintained in the composite. An efficiency of 4–6% on curcumin loading inside MGs is expected by both the elemental and TG analyses, which confirm the same results. 

Concerning the releases, different behaviors have been registered as a function of the medium: in SIF, the swelling is gradual, so it permits a controlled release that persists over time; tests conducted in SGF demonstrated that beads do not swell if they are put in contact with an extremely acidic environment (pH 1.2 of SGF), but the rehydration process takes place only in a slightly acid environment (pH 6.8 of SIF). Thanks to this phenomenon, we can assume a selective release at the intestinal level and not at the gastric one. These trends suggest a very interesting result: a specific release of curcumin (3 mg released in 4 h) in the intestinal tract and not in the gastric tract. Moreover, an increment of stability in the simulated gastrointestinal tract in shown by the triphasic systems (alginate, MGs, and curcumin) with respect to the MGs systems.

CMA tests confirm the well-known catalytic activity of the couple Ce^3+^/Ce^4+^ and the absence of catalytic behavior made by the contribution of curcumin.

Curcumin is very active in scavenging the DPPH radicals, and only very low concentrations prevent the complete radical reduction. Anti-radical action is practically absent when only MG is present. Analyzing the radical degradation during 24 h, a noticeable reducing action was also observed for cerium-containing samples. This suggests an independent effect of the metal ion, although it was lower than curcumin.

The amount of curcumin released varies from 0.8–1 μmol/g for MGs in 8 h to 3.0–3.1 μmol/100 mg for beads. For both systems, the release is sufficient to reach the antioxidant activity threshold. The results suggest that in MGs, the release follows a diffusive and erosive mechanism of the glass matrix, whereas, in the beads, initially curcumin is released on the surface, and then the release became diffusive and ruled by swelling of the polymeric lattice.

## 4. Material and Methods

### 4.1. General Procedures

All the chemicals and solvents were purchased with the highest purity grade available and used without further purification unless otherwise specified; pH measurements were carried out using a calibrated pH-meter (Hach).

UV–visible spectra were recorded using a JASKO V-570 UV/Vis/NIR spectrophotometer at 298 K in the 250–600 nm spectral range employing quartz cells (1 cm optical path). Elemental analysis was performed on a Thermo Scientific™ FLASH 2000 CHNS Analyzer. TG analyses were performed using a Seiko SSC 5200 in a temperature range between 25 °C and 1000 °C, with a heating rate of 1 °C/min. The morphology of the beads before and after soaking was examined by SEM using a JSM-6335F (JEOL) microscope operating at 20 kV. IR spectra were recorded in the 4000–400 cm^−1^ spectral range, using a FT-IR Jasco 4700 equipped with the ATR proONE.

### 4.2. Synthesis

In this work, MGs were synthesized following the sol–gel method by using the evaporation-induced self-assembly process (EISA) [1,2]. All the syntheses require a symmetric triblock copolymer comprising poly(ethylene oxide) (PEO) and poly(propylene oxide) (PPO), in an alternating linear fashion, and a non-ionic surfactant used as a structural agent, Pluronic^®^ P123 [3].

For this study, two different MGs were studied: starting from the composition of 80%SiO_2_–20%CaO (MG) and adding, to one sample, 5% of CeO_2_ (MG_5.0Ce). Each sample was prepared starting from the precursor of SiO_2_, CaO, and CeO_2_: tetraethyl orthosilicate (TEOS) (98%, Sigma Aldrich, Milan, Italy), calcium nitrate tetrahydrate Ca(NO_3_)_2_∙4H_2_O (99.5%, Sigma Aldrich), and cerium nitrate hexahydrate Ce(NO_3_)_3_∙6H_2_O (99%, Sigma Aldrich, Milan, Italy).

The theoretical molar compositions of the samples are reported in Table 7.

To conduct the syntheses [28,29], 2 g of Pluronic^®^ P123 was added to a solution composed of 80 mL of ethanol and 1 mL of hydrochloric acid 10%m/m. The solution was stirred at room temperature (RT) until complete dissolution. Then, for the synthesis of MG, 10 mL of TEOS and 1.99 g of Ca(NO_3_)_2_∙4H_2_O were added at RT in sequence, under continuous stirring, for approximately 3 h. For the synthesis of MG_5.0Ce, the amount of 1.38 g of Ce(NO_3_)_3_∙6H_2_O was also added. 

These solutions were stirred overnight to allow for the formation of a gel phase. Following that, they were transferred to Petri dishes for 48 h to undergo the EISA process. Successively, the dried gels were removed as homogeneous and transparent membranes and heated at 700 °C for 3 h under an air atmosphere to remove the surfactant and nitrate groups and to stabilize the resultant mesoporous glasses. The powders were obtained by grinding the mesoporous glass pieces with an agate pestle. The powders were sieved to a particle size < 125 µm and stored in a desiccator until further use.

The in vitro bioactivity of B-MG and powdery MGs (P-MG) was confirmed in previous papers [4,5,32]. To evaluate drug release both in pure distilled water and physiological environment, a simulated body fluid (SBF) buffered at a pH of 7.4 according to Kokubo was prepared [6,33]. Table 8 shows the comparison between plasma and SBF ion concentrations (expressed as mM).

### 4.3. Curcumin Loading onto P-MGs

For the two bioactive glass samples prepared in the previous paragraph, curcumin loadings were performed by using the impregnation method: an ethanol solution of curcumin (98%, Sigma Aldrich; 40 mL, 5 mg/mL) was kept in contact with 1 g of sample (grain size < 125 µm) at RT for 48 h to obtain 1–2% m/m of curcumin for each glass (P-MG_curc5) [34,35]. Loadings were performed on different MGs: the unaltered MG (P-MG_curc5) and the cerium-doped one (P_MG_5.0Ce_curc5). A blank sample was also prepared in identical conditions using only ethanol as a contact solution. 

The final solutions were then filtered, and the solids were dried for 24 h at 70 °C.

### 4.4. P-MGs Curcumin Content Determination

Curcumin content was determined through solvent extraction followed by a UV-Vis determination. The data were reported in Table 1.

Then, 250 mg of the solid was put in contact with 25 mL of ethyl acetate for 3 h, and the curcumin released from the solid was determined spectrophotometrically at 420 nm using a calibration curve of curcumin in ethyl acetate in the range of 5·10^−6^–2·10^−5^ M [35,36].

### 4.5. Beads’ Synthesis

Alginate beads were synthesized following the cross-linking ionic method: starting from sodium alginate solution, through an ion exchange between sodium and calcium present in a second solution, a calcium alginate lattice was formed [37].

Under continuous stirring, 0.5 g of the unloaded glass powder samples (P-MG and P-MG_5.0Ce, grain size < 125 µm) were added to 20 mL of sodium alginate solution 1%m/m until the formation of a homogeneous suspension. The suspension was added dropwise into the solution of calcium chloride 0.1 M. The formed beads (B-MG and B-MG_5.0Ce) were kept under continuous stirring overnight to allow for the complete digestion and the formation of a resistant lattice of calcium alginate. Under continuous stirring, 0.5 g of the loaded glass powder samples (P-MG_curc5 and P-MG_5.0Ce_curc5) were added to 40 mL of ethanol solution of curcumin (5 mg/mL) and 20 mL of sodium alginate solution 1%m/m until the formation of a homogeneous suspension. The suspension was added dropwise into the solution of calcium chloride 0.1 M. The formed beads (B-MG_curc5 and B-MG_5.0Ce_curc5) were kept under continuous stirring overnight to allow for the complete digestion and the formation of a resistant lattice of calcium alginate.

For comparison purposes, 20 mL of sodium alginate solution 1%m/m was added dropwise into the solution of calcium chloride 0.1 M. The formed beads (B) were kept under continuous stirring overnight to allow for the complete digestion and the formation of a resistant lattice of calcium alginate. Moreover, a mix of 20 mL of sodium alginate solution 1%m/m and 40 mL of ethanol solution of curcumin (5 mg/mL) was added dropwise into the solution of calcium chloride 0.1 M. The formed beads (B-curc) were kept under continuous stirring overnight to allow for the complete digestion and the formation of a resistant lattice of calcium alginate.

After that, the beads were filtered under vacuum and washed with ethanol. Thermal treatment at 60 °C for removing water was then performed.

### 4.6. Catalase Mimetic Activity (CMA) Tests

The catalase mimetic activity tests (CMA) were performed to evaluate enzymatic-like activity of samples. 

To conduct the test, a solution 1 M of H_2_O_2_ was added to the powder glass sample using a ratio MG/H_2_O_2_ of 5 mg/mL. In addition, the blank reference, i.e., only the solution of H_2_O_2_, was prepared. Each sample was incubated at 37 °C and 120 rpm for five different time periods: 1 day, 4 days, 5 days, 6 days, and 7 days. At the end of incubation, the solutions were filtered, and the filtrates were collected. To perform CMA, 10 mL of filtrate was diluted 1:10, and 10 mL of the diluted solution was added to 20 mL of deionized water and 20 mL of sulfuric acid 1:4. The titration was performed using KMnO₄ 0.1 N, previously standardized. 

### 4.7. 2,2-Diphenyl-1-Picrylhydrazyl (DPPH) Test

This test allows for the evaluation of the antioxidant activity through a spectrophotometric method by using a DPPH (99.9% Sigma Aldrich) solution. DPPH presents a characteristic adsorption peak at 517 nm, but when an oxidant agent is present in the solution, a decrease of the adsorption is registered. 

A solution 1.2·10^−2^ M of DPPH in methanol was prepared and then diluted 1: 200. 

Different amounts of samples were used: 10 mg, 20 mg, 30 mg, 50 mg, 70 mg, and 100 mg. All samples were added to 10 mL of DPPH 6 × 10^−5^ M and incubated at 37 °C, 120 rpm, in the dark for 2 h. Simultaneously, a blank solution of only DPPH was prepared and incubated. After incubation, the solutions were centrifuged, and the supernatants were collected and analyzed spectrophotometrically. At the same time, to evaluate the antioxidant activity of curcumin, assuming a loading of 15% of curcumin inside MG and a complete release of it in solution, an analogous solution (10^−2^ M in methanol) was prepared. 

### 4.8. DPPH Degradation Kinetic

To determine the DPPH degradation kinetic constant, a solution with the same concentration as the previous one was prepared (0.33 g of P-MG in 1 mL) but different timing was studied: 1 h, 2 h, 3 h, 4 h, 5 h, 6 h, 7 h, 8 h, and 24 h. In addition, a reference solution of curcumin was prepared (0.50 mg of curcumin in 10 mL of a DPPH solution 6∙10⁻^5^ M).

Determining the radical scavenging effect (RSE%), following the Gulcin equation [3], it is possible to evaluate the action against radicals.

### 4.9. Swelling Test

The swelling test was used to evaluate the beads’ rehydration rate and degradation. The rehydration test is performed in different mediums: H_2_O dd, SBF, D-MEM (Dulbecco’s Modified Eagle Medium), SGF (simulated gastric fluid), and SIF (simulated intestinal fluid). 

In a multi-well plate, an appropriate amount of the beads was added together with 2 mL of the biological fluid. The plate was incubated at 37 °C and 120 rpm. At different timing, according to the biological fluid, beads were collected, dried, weighed, and re-immersed into the swelling medium. The weight variation was used to evaluate the rehydration rate, according to Equation (4) [38,39].

(4) $$\%\;W\;rehydration=100*\frac{W_{t}-W_{d}}{W_{d}}\;$$

where *W_t_* is rehydrated bead weight, *t* is the time, and *W_d_* is the initial weight.

### 4.10. Curcumin Release

For these tests, 50 mg of the sample was put in contact with 5 mL of the appropriate medium (SBF, SGF, or SIF) for different time periods: 0 min, 30 min, 1 h, 2 h, 3 h, 4 h, 5 h, 6 h, 7 h, 8 h, and 24 h. For the analysis, a limpid released solution of curcumin is mandatory; therefore, the suspension was centrifuged, and the supernatant was collected, then the solid sample was re-suspended in a new amount of medium. After centrifugation, 3 mL of the supernatant was extracted with the mixture ethylacetate: methanol 95:5 V:V. The amount of curcumin released was determined after its extraction from the medium by using UV-Vis spectroscopy (concentration of calibration solutions: 2 µM–50 µM). 

Samples treated in SGF for 4 h were washed with deionized water and then used for release in SIF [7].

## 5. Conclusions

In light of the results obtained, it can be concluded that the investigated scaffolds represent multifunctional systems potentially useful in drug delivery. One of the main achievements is the improvement in curcumin stabilization and bioavailability, especially in the intestine, where it can exert its therapeutic action, mainly against colorectal cancer. The fundamental release behavior of hybrid materials has been shown and demonstrated.

In conclusion, all scaffolds take advantage of the synergic effect of cerium and curcumin, and they can be used as drug delivery systems for curcumin, with high release.

## Figures and Tables

**Table 1:** Theoretical and experimental compositions of the samples synthesized *.

Sample	%m/m Alginate		%m/m Glass		%m/m Curcumin	
	Theoretical	Experimental	Theoretical	Experimental	Theoretical	Experimental
P-MG_curc5	-	-	83.3	98.2	16.7	1.8
P-MG_5.0Ce_curc5	-	-	83.3	98.4	16.7	1.6
B	100.0	99.1	-	-	-	-
B-curc	95.0	93.8	-	-	5.0	6.2
B-MG	28.6	36.6	71.4	63.4	-	-
B-MG_curc5	26.7	27.4	66.7	68.4	6.6	4.2
B-MG_5.0Ce	28.6	39.3	71.4	60.7	-	-
B-MG_5.0Ce_curc5	26.7	26.8	66.7	67.1	6.6	6.1

* The experimental %m/m Glass was determined by TG-thermogravimetry, while %m/m Curcumin was determined by extraction, as reported in Section 4.3.

**Table 2:** Average dimensions of synthesized beads.

Sample	Average Diameter (mm)
B	1.4 ± 0.2
B-MG	1.8 ± 0.1
B-MG_curc5	2.3 ± 0.1
B-MBG_5.0Ce	2.0 ± 0.1
B-MBG_5.0Ce_curc5	2.4 ± 0.1

**Table 3:** Linear equations and kinetic order of degradation in for each sample.

2° Order			1° Order		
**H_2_O_2_**	y = 0.0041x + 1.0065	R^2^ = 0.846	**H_2_O_2_**	y = 0.0052x	R^2^ = 0.743
**P-MG**	y = 0.0174x + 1.0042	R^2^ = 0.960	**P-MG**	y = 0.0172x	R^2^ = 0.9577
**P-MG_curc5**	y = 0.0210x + 1.0247	R^2^ = 0.922	**P-MG_curc5**	y = 0.0238x	R^2^ = 0.8425
**P-MG_5.0Ce**	y = 0.8123x + 0.2239	R^2^ = 0.7301	**P-MG_5.0Ce**	y = 0.2564x	R^2^ = 0.944
**P-MG_5.0Ce_curc5**	y = 0.9437x + 0.0679	R^2^ = 0.7154	**P-MG_5.0Ce_curc5**	y = 0.2741x	R^2^ = 0.948

* Kinetic orders (2° or 1°) equations with higher R^2^ are highlighted in grey.

**Table 4:** Linear equations and kinetic order of degradation in each sample.

1° Order			2° Order		
**B-MG**	y = 0.0119x	R^2^ = 0.9195	**B-MG**	y = 0.0138x + 0.9925	R^2^ = 0.9296
**B-MG_curc5**	y = 0.0226x	R^2^ = 0.7887	**B-MG_curc5**	y = 0.0228x + 1.0086	R^2^ = 0.7994
**B-MG_5.0Ce**	y = 0.278x	R^2^ = 0.9858	**B-MG_5.0Ce**	y = 0.9324x + 0.3377	R^2^ = 0.8911
**B-MG_5.0Ce_curc5**	y = 0.361x	R^2^ = 0.9838	**B-MG_5.0Ce_curc5**	y = 1.8335x − 0.4935	R^2^ = 0.852

* Kinetic orders (2° or 1°) equations with higher R^2^ are highlighted in grey.

**Table 5:** Radical scavenging effect of some samples tested to evaluate the antioxidant power against the radical DPPH.

Concentration mg/mL	RSE%	Concentration mg/mL	RSE%	Concentration mg/mL	RSE%
P-MG 1 mg/mL	8	P-MG_5.0Ce 1 mg/mL	0	Curc 5∙10⁻^3^ mg/mL	78
P-MG 5 mg/mL	8	P-MG_5.0Ce 5 mg/mL	7	Curc 3.3∙10⁻^3^ mg/mL	64
P-MG 10 mg/mL	7	P-MG_5.0Ce 10 mg/mL	14	Curc 1.7∙10⁻^3^ mg/mL	41
P-MG_curc5 1 mg/mL	15	P-MG_5.0Ce_curc5 1 mg/mL	25		
P-MG_curc5 5 mg/mL	50	P-MG_5.0Ce_curc5 5 mg/mL	44		
P-MG_curc5 10 mg/mL	100	P-MG_5.0Ce_curc5 10 mg/mL	100		

**Table 6:** Linear equations and related parameters for the kinetic study of antioxidant power in the DPPH test.

Sample	Linear Equations	
DPPH 6∙10⁻^5^ M	y = 0.0705x + 3.981	R^2^ = 0.9929
P-MG	y = 0.078x + 4.0149	R^2^ = 0.9875
P-MG_5.0Ce	y= 0.0791x + 4.051	R^2^ = 0.9591
P-MG_curc5	y = 0.0962x + 3.3147	R^2^ = 0.9315
P-MG_5.0Ce_curc5	y = 0.165x + 3.3016	R^2^ = 0.9633
Curc	y = 0.7276x + 5.0969	R^2^ = 0.7704

**Table 7:** Theoretical molar composition of bioactive glasses.

Glass	SiO_2_ (mol%)	CaO (mol%)	CeO_2_ (mol%)
MG	80	20	-
MG_5.0Ce	80	15	5

**Table 8:** Comparison between the composition (mM) of human plasma and simulated body fluid (SBF).

	Na^+^	K^+^	Mg^2+^	Ca^2+^	Cl^−^	HCO_3_^−^	HPO_4_^2−^	SO_4_^2−^	pH
**SBF**	142.0	5.0	1.5	2.5	147.8	4.2	1.0	0.5	7.4
**Plasma**	142.0	5.0	1.5	2.5	103.0	27.0	1.0	0.5	7.20/7.40

## Data Availability

Additional data that support the findings of this study are available from the corresponding author.
